# Deep Brain Stimulation as a Rehabilitation Amplifier: A Precision-Oriented, Network-Guided Framework for Functional Restoration in Movement Disorders

**DOI:** 10.3390/jcm15020492

**Published:** 2026-01-08

**Authors:** Olga Mateo-Sierra, Beatriz De la Casa-Fages, Esther Martín-Ramírez, Marta Barreiro-Gómez, Francisco Grandas

**Affiliations:** 1 Department of Neurosurgery, Gregorio Marañón University Hospital, C/Dr Esquerdo 46, 28007 Madrid, Spain; 2 Gregorio Marañón Research Institute, Gregorio Marañón University General Hospital, C/Dr Esquerdo 46, 28007 Madrid, Spain; 3 Department of Surgery, Medicine School, Complutense University of Madrid, 28040 Madrid, Spain; 4 Movement Disorders Unit, Department of Neurology, Gregorio Marañón University Hospital, C/Dr Esquerdo 46, 28007 Madrid, Spain; 5 Department of Radiology, Gregorio Marañón University Hospital, C/Dr Esquerdo 46, 28007 Madrid, Spain; 6 Department of Rehabilitation, Gregorio Marañón University Hospital, C/Dr Esquerdo 46, 28007 Madrid, Spain; 7 Department of Medicine, Medicine School, Complutense University of Madrid, 28040 Madrid, Spain

**Keywords:** deep brain stimulation, connectomics, movement disorders, Parkinson’s disease, essential tremor, dystonia, adaptive neuromodulation, rehabilitation, tractography, sensing-enabled DBS

## Abstract

**Background**: Deep brain stimulation (DBS) is increasingly understood as a precision-oriented neuromodulation therapy capable of influencing distributed basal ganglia–thalamo–cortical and cerebellothalamic networks. Although its symptomatic benefits in Parkinson’s disease, essential tremor, and dystonia are well established, the extent to which DBS supports motor learning, adaptive plasticity, and participation in rehabilitation remains insufficiently defined. Traditional interpretations of DBS as a focal or lesion-like intervention are being challenged by electrophysiological and imaging evidence demonstrating multiscale modulation of circuit dynamics. **Objectives and methods**: DBS may enhance rehabilitation outcomes by stabilizing pathological oscillations and reducing moment-to-moment variability in motor performance, thereby enabling more consistent task execution and more effective physiotherapy, occupational therapy, and speech–language interventions. However, direct comparative evidence demonstrating additive or synergistic effects of DBS combined with rehabilitation remains limited. As a result, this potential is not fully realized in clinical practice due to interindividual variability, limited insight into how individual circuit architecture shapes therapeutic response, and the limited specificity of current connectomic biomarkers for predicting functional gains. **Results:** Technological advances such as tractography-guided targeting, directional leads, sensing-enabled devices, and adaptive stimulation are expanding opportunities to align neuromodulation with individualized circuit dysfunction. Despite these developments, major conceptual and empirical gaps persist. Few controlled studies directly compare outcomes with versus without structured rehabilitation following DBS. Heterogeneity in therapeutic response and rehabilitation access further complicates the interpretation of outcomes. Clarifying these relationships is essential for developing precision-informed frameworks that integrate DBS with rehabilitative strategies, recognizing that current connectomic and physiological biomarkers remain incompletely validated for predicting functional outcomes. **Conclusions**: This review synthesizes mechanistic, imaging, and technological evidence to outline a network-informed perspective of DBS as a potential facilitator of rehabilitation-driven functional improvement and identifies priorities for future research aimed at optimizing durable functional restoration.

## 1. Introduction

Essential tremor (ET), Parkinson’s disease (PD), and dystonia are the most prevalent chronic movement disorders and major contributors to long-term motor disability worldwide [1,2,3,4]. Their global prevalence continues to rise, driven largely by population aging and increased life expectancy, underscoring the need for therapeutic strategies that sustain long-term functional independence [5,6,7]. At the same time, the evidence base supporting advanced neuromodulatory and rehabilitation-integrated interventions remains unevenly distributed across healthcare systems and populations. This growing burden highlights the limitations of purely symptomatic approaches and reinforces the importance of interventions that not only control motor features but also enhance functional capacity. While symptom reduction is a prerequisite for functional improvement, gains in daily functioning, independence, and participation often require stabilization of motor performance and engagement in task-specific rehabilitation beyond symptomatic control alone.

Deep brain stimulation (DBS) has become the most effective neurosurgical therapy for medication-refractory movement disorders, marking a transition from irreversible ablative procedures to reversible and adjustable neuromodulation [8,9,10,11]. Early interpretations framed DBS as a “functional lesion,” reflecting its ability to suppress pathological activity within targeted nuclei [12,13]. However, accumulating electrophysiological, neurochemical, and imaging evidence has reframed DBS as a network-level intervention, acting through modulation of distributed motor circuits rather than by focal suppression at the stimulation site [14,15,16,17]. Recent advances in connectomics have further highlighted the limitations of classical nucleus-centric models, showing that DBS outcomes are shaped not solely by electrode proximity to anatomical targets but by the broader patterns of circuit engagement and network reorganization enabled through stimulation [18,19,20]. This shift from anatomical to systems-based reasoning challenges traditional assumptions about target selection, mechanisms of action, and the therapeutic reach of DBS.

Despite these mechanistic and technological advances, DBS remains underutilized as a partner to structured rehabilitation, even though neuromodulation can provide a stable physiological substrate for task-specific training, motor learning, and long-term skill acquisition [21,22,23]. By stabilizing motor fluctuations and reducing performance variability, DBS may enable patients to participate more effectively in physiotherapy, occupational therapy, and speech–language interventions. Viewed in this context, DBS may create neural conditions that support adaptive plasticity and facilitate engagement with structured rehabilitation, thereby contributing to more durable functional restoration. The purpose of this review is to synthesize mechanistic concepts, imaging frameworks, and technological innovations that support a precision-oriented, network-based integration of DBS with multidisciplinary rehabilitation. We outline the evolution from lesion-based and nucleus-centric models to modern connectomic paradigms; examine phenotypic and circuit-level heterogeneity; and discuss how DBS, when combined with structured rehabilitation, can optimize motor performance, functional independence, and long-term outcomes in movement disorders. In this context, ‘precision’ refers to an emerging, circuit-informed approach to neuromodulation rather than a fully validated paradigm for individualized outcome prediction.

## 2. Materials and Methods

### 2.1. Literature Search Strategy and Scope of the Review

This review synthesizes mechanistic, imaging, technological, and clinically relevant studies, defined as investigations reporting functional, behavioral, or rehabilitation-related outcomes in DBS-treated patients. Literature was identified through searches in PubMed, Scopus, and Web of Science up to December 2025 using combinations of the terms “deep brain stimulation,” “motor learning,” “plasticity,” “connectomics,” “rehabilitation,” “adaptive DBS,” “tractography-guided targeting,” and disorder-specific terms including “Parkinson’s disease,” “essential tremor,” and “dystonia.” Reference lists of key publications were examined to supplement database searches.

This review focuses on rehabilitation as functional motor retraining in the context of movement disorders treated with DBS. Rehabilitation-related examples discussed in the manuscript are drawn from the existing literature on Parkinson’s disease, essential tremor, and dystonia, rather than from post-stroke, traumatic, or other non–non-movement-disorder indications.

No language or date restrictions were applied, although priority was given to mechanistic, imaging, and clinically relevant studies published within the last decade alongside foundational historical work. The majority of included mechanistic and imaging studies were published within the last decade, while earlier foundational studies were retained to provide historical and conceptual context.

As this is a conceptual narrative review, no systematic screening, risk-of-bias assessment, or meta-analysis was performed. Studies were selected for relevance to (i) multiscale DBS mechanisms; (ii) imaging supporting circuit-level targeting; (iii) technological innovations in neuromodulation; and (iv) rehabilitation approaches intersecting with DBS. Although no formal risk-of-bias assessment was performed, small pilot studies and isolated case reports were not used to support efficacy claims and were considered primarily for hypothesis generation or proof-of-concept.

This review is based solely on previously published studies and does not involve any new research with human participants or animals.

### 2.2. Population Representativeness and Scope Considerations

Although this narrative review did not systematically extract demographic or socioeconomic variables from the included studies, it is important to acknowledge that the current evidence base supporting DBS—particularly mechanistic, imaging, and rehabilitation-related research—derives predominantly from high-income Western academic centers. Consequently, the concepts discussed in this review reflect the populations currently represented in the literature.

## 3. Integrated Framework for Network-Level DBS Mechanisms and Clinical Translation

### 3.1. Epidemiology and Global Burden of Movement Disorders

**Figure 1:**
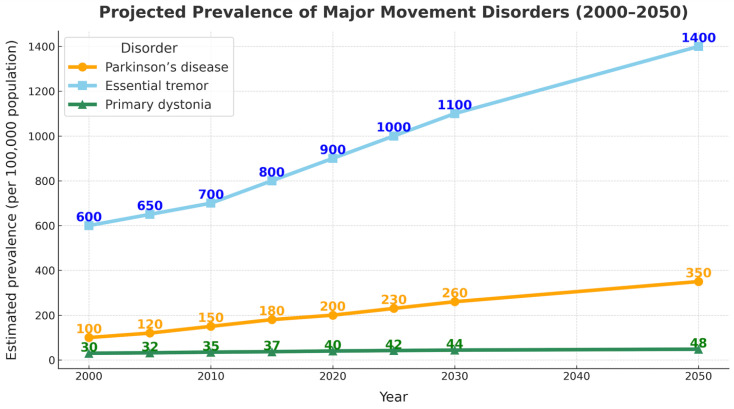
Projected prevalence of major movement disorders (2000–2050). Values reflect approximate population-level trends derived from published epidemiological data. These projections illustrate the growing global burden of movement disorders and underscore the need for therapeutic strategies that enhance functional capacity and reduce long-term disability.

ET, PD, and dystonia are among the most common chronic movement disorders and together represent substantial long-term sources of disability (Figure 1). ET affects approximately 4–5% of adults over 65 years of age, underscoring its significant public-health impact in aging populations [1]. The global prevalence of PD is projected to surpass 12 million by 2040, largely driven by demographic aging and increased life expectancy [2]. Dystonia represents the third most frequent movement disorder encountered in tertiary care, encompassing focal, segmental, generalized, and genetic forms with heterogeneous clinical trajectories [3,4]. Although considerably less prevalent, rare and highly disabling dystonic phenotypes continue to be reported, and emerging case-based evidence suggests the potential for DBS to restore meaningful functional capacity even in exceptional or atypical presentations [24,25].

Beyond their cardinal motor manifestations, these conditions impose substantial functional, psychosocial, and socioeconomic burdens. Global modeling indicates a sustained rise in PD-associated disability, reflecting both increased prevalence and progression-related morbidity [5,6,7]. ET, long mischaracterized as a benign disorder, is now recognized as a major contributor to functional dependence, reduced quality of life, and increased healthcare utilization [26]. Although less frequently quantified in burden-of-disease frameworks, dystonia also contributes significantly to disability-adjusted life years, chronic pain, vocational limitations, and long-term care needs [27].

These epidemiological trends emphasize the need for therapeutic strategies that move beyond symptom suppression to restore functional capacity and support sustained independence. As disease prevalence increases and survival improves, the cumulative burden of long-term disability, dependence, and participation restrictions becomes a dominant determinant of quality of life, underscoring the need for strategies that extend beyond symptom suppression alone. When embedded within structured rehabilitation paradigms, DBS offers an opportunity to modulate pathological networks and enhance adaptive plasticity, thereby promoting more durable improvements in daily functioning.

Notably, it should be recognized that the evidence informing network-guided and rehabilitation-integrated DBS frameworks is derived from a limited range of populations, which has implications for the external validity of precision-oriented approaches.

### 3.2. Historical Evolution of DBS Concepts

#### 3.2.1. From Lesion-Based Surgery to Reversible Neuromodulation

**Figure 2:**
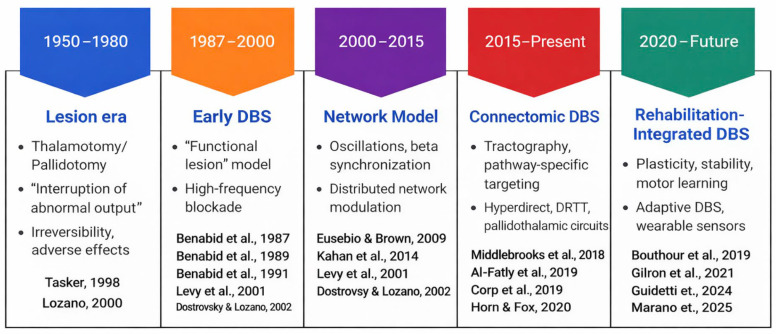
Conceptual evolution of deep brain stimulation (DBS). Five major eras illustrate the progression from lesion-based interventions to modern, network-guided neuromodulation. Early irreversible lesioning was replaced by high-frequency DBS, later reinterpreted within network-level frameworks. Advances in connectomics enabled pathway-specific targeting, and from 2020 onward, sensing-enabled and adaptive DBS technologies have driven the transition toward rehabilitation-integrated neuromodulation. Tasker; Lozano [10,11]; Benabid [8,9,28]; Levy; Dostrovsky [8,9,12,13,28]; Eusebio [29]; Kahan; Fasano [30,31]; Middlebrooks; Al-Fatly; Corp; Horn [16,17,18,19]; Bouthour; Gilron; Guidetti; Marano [32,33,34,35].

The development of DBS marked a major shift from irreversible ablative procedures toward adjustable neuromodulation (Figure 2). Lesioning techniques such as thalamotomy and pallidotomy demonstrated that interrupting aberrant basal ganglia signaling could reduce tremor, rigidity, and dyskinesias, but concerns regarding irreversibility and adverse effects limited their widespread use [10,11].

The introduction of high-frequency stimulation by Benabid and colleagues in the late 1980s was transformative. Ventral intermediate nucleus (VIM) stimulation reproduced the therapeutic effects of lesions while preserving reversibility and allowing postoperative titration [8,28]. This approach rapidly expanded to Parkinson’s disease (PD), establishing DBS as a viable alternative to lesioning [9].

Early mechanistic interpretations framed DBS as a “functional” or “adjustable lesion,” reflecting its capacity to suppress pathological activity within targeted nuclei [12,13]. Subsequent electrophysiological evidence, however, demonstrated that high-frequency stimulation does not simply silence local neuronal activity. Instead, it imposes a structured exogenous drive that disrupts pathological oscillatory dynamics [14]. This transition from a lesion-based to a network-based explanatory model began to suggest that clinical benefits extend beyond focal inhibition.

#### 3.2.2. Nucleus-Centric DBS and Emerging Complexity

**Figure 3:**
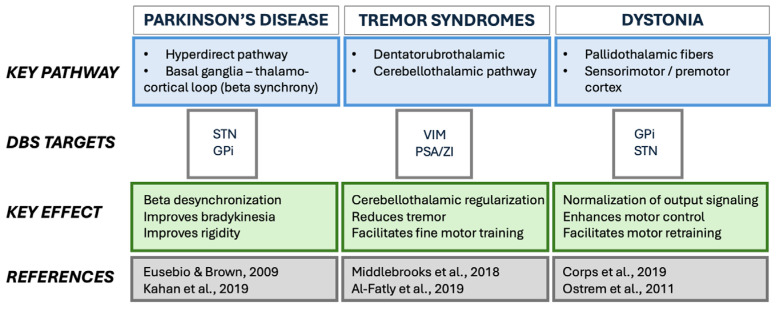
Pathways, DBS targets, and key physiological effects across movement disorders. Schematic overview of dominant dysfunctional circuits, preferred DBS targets, and associated physiological effects across movement disorders. Eusebio & Brown P [29], Kahan [30], Middlebrooks [18], Al-Fatly [16], Corps [19], Ostrem [42].

Classical DBS strategies aligned each movement disorder with a canonical target—VIM for essential tremor, the subthalamic nucleus (STN) for PD, and the globus pallidus internus (GPi) for dystonia [36,37], as depicted in Figure 3. This nucleus-based model produced major therapeutic advances, with randomized trials confirming durable improvements across tremor, bradykinesia, rigidity, and dystonia [31,38,39,40,41].

As clinical experience grew, several observations challenged this framework. Physiological responses to stimulation proved more complex than simple suppression, with heterogeneous axonal and somatic effects extending beyond the stimulation site [43,44,45]. Phenotypic variability within ET, PD, and dystonia complicated attempts to generalize stimulation responses across patients and phenotypes [4,18,46].

Additionally, early safety concerns regarding MRI in patients with implanted devices limited high-resolution characterization of stimulation-induced changes [47,48]. The subsequent development of device-specific MRI protocols enabled higher-resolution interrogation of stimulation-related network effects [49], solidifying the conceptual transition from a focal intervention toward a therapy that exerts its benefits by engaging distributed motor circuits.

### 3.3. Phenotypes and Network Level Heterogeneity

Clinical experience with DBS rapidly demonstrated that major movement disorders exhibit substantial phenotypic heterogeneity, reflected in the variable engagement of basal ganglia–thalamo–cortical and cerebellothalamic networks (Table 1). In PD, tremor-dominant, akinetic–rigid, postural instability/gait disorder (PIGD), and cognitively vulnerable subtypes show distinct circuit dependencies that shape therapeutic response [46,50]. ET and dystonia show comparable diversity, with symptom profiles reflecting differential involvement of motor, cerebellar, and associative pathways [4,51].

Similar clinical symptom scores may arise from distinct underlying circuit dysfunctions, implying that patients with comparable motor severity may differ substantially in their neural substrates and, consequently, in their responsiveness to both stimulation and rehabilitation.

Electrophysiological and imaging evidence increasingly supports the view that these conditions represent spectra of circuit dysfunction [15,44]. Tremor severity aligns with cerebellothalamic pathways—especially the dentato-rubro-thalamic path (DRTT)—[16,23], whereas bradykinesia and rigidity relate to hyperdirect cortico–subthalamic projections, and cognitive vulnerability maps onto associative–limbic networks [21,52,53]. In dystonia, responsiveness to GPi stimulation corresponds to pallidothalamic and sensorimotor network engagement [19,40,54,55].

Network specificity also explains therapeutic variability within tremor syndromes; while classic ET responds to VIM stimulation, ET-plus and cerebellar variants may benefit from posterior subthalamic area (PSA) or zona incerta (ZI) targeting, where cerebellothalamic fibers converge [16,18]. Dystonic tremor, reflecting hybrid cerebellothalamic–pallidal involvement, often requires individualized targeting [56]. Rare dystonic presentations, including GA1-related and KMT2B-associated dystonia, can improve meaningfully with GPi-DBS, though outcomes remain variable across syndromes [24,25].

Overall, these findings, summarized in Table 1, support the concept that identifying the dominant dysfunctional circuit—cerebellothalamic, hyperdirect, pallidothalamic, or associative–limbic—can inform individualized DBS strategies and is associated with improved clinical outcomes, as illustrated in Figure 3.

### 3.4. Local Effects and Multiscale Biological Mechanisms

Building on these network-level distinctions, mechanistic insights into DBS derive from studies of STN stimulation in PD, where electrophysiological and cellular responses have been characterized in greatest detail (Table 2). At the microscale, DBS preferentially activates large myelinated axons while suppressing intrinsic somatic firing, generating orthodromic and antidromic signals that reshape information flow across basal ganglia circuits [12,45]. High-frequency stimulation introduces a more regular firing pattern that counteracts pathological bursting and excessive beta synchrony, reducing moment-to-moment variability in motor output [57,58].

Local responses differ across targets. Human recordings demonstrate that STN stimulation rapidly suppresses intrinsic firing, VIM stimulation evokes a brief activation burst before silencing, and GPi stimulation produces transient inhibition followed by recurrent reactivation—patterns reflecting the unique convergence of afferent and efferent connections at each site [44]. At the mesoscale, DBS suppresses pathological beta oscillations, induces short-latency cortico–subcortical entrainment, and evokes resonant neural activity (ERNA), a high-frequency signature whose amplitude scales with stimulation intensity and differs between STN and GPi [44]. These oscillatory dynamics represent a physiological bridge between local neuronal effects and broader functional improvements, consistent with macroscale network modulation summarized in Table 2.

Beyond neuronal elements, DBS also modulates non-neuronal components of the microenvironment. Astrocytes regulate neurotransmission through activity-dependent glutamate and adenosine release, influence extracellular matrix signaling, and may foster conditions that support plasticity. Electrode implantation and chronic stimulation additionally induce glial and epigenetic adaptations—including reductions in astrocytic reactivity and changes in DNA methylation and microRNA expression—that may contribute to long-term functional stability [43].

**Figure 4:**
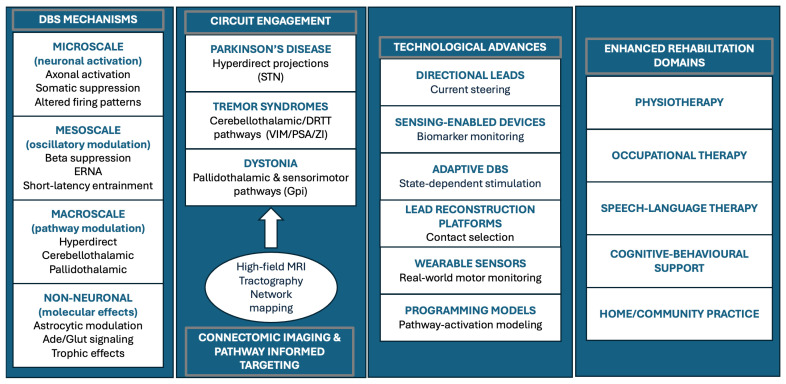
Integrative overview of DBS mechanisms, disease-specific circuit engagement, key technological advances, and rehabilitation domains. Connectomic imaging and pathway-informed targeting link multiscale neuromodulation effects with clinical and rehabilitative applications. The arrow denotes the contribution of high-field MRI tractography and network mapping to circuit-informed DBS targeting.

Together, these multiscale responses illustrate that DBS operates through layered mechanisms encompassing cellular, oscillatory, and network domains. Although not yet indicative of a definitive disease-modifying effect, this multilevel modulation provides a neurophysiological foundation upon which rehabilitation can build by promoting more consistent motor performance and enhancing the capacity for adaptive plasticity, an alignment illustrated in Figure 4, which outlines the principal rehabilitation goals after DBS.

### 3.5. Imaging Evidence for Network-Level Mechanisms

Imaging studies have been central to reframing DBS mechanisms. Before MRI protocols compatible with implanted hardware became available, PET and SPECT provided the first in vivo evidence that stimulation induces metabolic and perfusion changes in regions distant from the electrode site—including motor cortex, cerebellum, thalamus, and basal ganglia—supporting a circuit-level interpretation of DBS effects [48,59]. Although limited by radiation exposure and temporal resolution, these modalities established the foundational concept that DBS modifies activity across broader motor systems.

The advent of device-specific MRI protocols enabled higher-resolution characterization of these network effects [49] (Table 1). Structural MRI and diffusion tractography studies have shown that clinical outcomes are more strongly associated with stimulation of symptom-relevant pathways than with proximity to nuclear boundaries [55,60,61]. These findings reinforce previously outlined symptom–circuit relationships, suggesting that pathway-level alignment is more closely associated with therapeutic response than anatomical proximity alone.

Functional imaging further supports this distributed-network perspective [21]. Resting-state fMRI identifies connectivity signatures associated with differential DBS responsiveness—stronger cerebellothalamic coupling in tremor syndromes and restoration of cortico–subcortical flexibility in PD [30,62]. In dystonia, intact GPi connectivity with premotor and cingulate regions has been associated with faster and more durable clinical improvement [19,55,63].

Imaging studies, including longitudinal and state-dependent designs, suggest that DBS is associated with progressive reorganization of motor networks rather than fixed, stimulation-dependent effects. STN stimulation alters cortico–subcortical communication patterns in PD, GPi stimulation normalizes sensorimotor network architecture in dystonia, and VIM/PSA stimulation modulates cerebellothalamic pathways in tremor syndromes, with tract involvement correlating with sustained symptom reduction [16,63,64]. Experimental work further suggests that thalamic stimulation may facilitate specific aspects of motor sequence learning in controlled experimental settings [65].

Collectively, these imaging findings support a network-level interpretation of DBS effects, while underscoring that most evidence remains associative rather than causal.

### 3.6. Surgical and Technological Advances Enabling Precision DBS

Advances in imaging, computational planning, and implantable hardware have progressively shifted DBS from a nucleus-based therapy to a circuit-informed, increasingly precision-oriented neuromodulation approach (Table 3). High-field MRI now enables reliable visualization of subcortical anatomy, while diffusion tractography provides patient-specific reconstructions of pathways relevant to tremor, bradykinesia, rigidity, and dystonia [15,16,18,19,61]. These modalities support targeting strategies that align electrode placement with symptom-relevant fiber trajectories and reduce reliance on indirect anatomical landmarks [47,49].

Trajectory planning has also evolved with the integration of multimodal image fusion, tractography constraints, and vascular avoidance algorithms, enabling safe implantation while maximizing engagement of intended therapeutic structures [18,49,61]. Intraoperative workflows increasingly incorporate imaging-guided verification or microelectrode recording, offering complementary methods to confirm lead positioning and reducing operative variability [66,67,68].

Hardware innovations have expanded the capacity to tailor stimulation to individual network architecture (Table 3) [69]. Directional leads allow current steering away from structures associated with side effects and toward pathways linked to therapeutic benefit, effectively broadening the clinical stimulation range [70,71]. Sensing-enabled pulse generators can record neural biomarkers such as beta bursts or tremor-related oscillations, providing objective data for programming and chronic physiological assessment [15,33,72]. These systems form the basis for adaptive (closed-loop) DBS, in which stimulation continuously adjusts according to real-time neural states, improving gait and tremor stability and reducing unnecessary energy delivery.

Modern programming platforms incorporate postoperative lead reconstructions and tractography-based pathway models, enabling clinicians to visualize which contacts most effectively engage the patient’s dominant dysfunctional circuit [15,73]. This approach supports efficient, rational programming while facilitating alignment of stimulation with individual functional goals.

### 3.7. Rehabilitation-Integrated DBS: Towards Network Restoration

DBS may facilitate rehabilitation by stabilizing neural dynamics and reducing moment-to-moment motor variability—conditions essential for motor learning [14,72,74]. This physiological regularization enhances responsiveness to physiotherapy and improves task-specific retraining efficiency, partly by reducing motor variability (Table 4) [34]. Clinically, this may translate into improved capacity for locomotor training, including gait and balance-focused interventions. This clinical effect aligns with recent mechanistic summaries demonstrating that DBS restores more physiologically regular network activity and supports functional performance [75].

While network stabilization provides a plausible physiological substrate for rehabilitation, mechanistic plausibility should not be conflated with demonstrated additive or synergistic clinical benefit, as controlled comparisons of DBS combined with rehabilitation versus DBS alone remain limited.

From a practical perspective, access to structured rehabilitation after DBS varies substantially across healthcare systems and socioeconomic contexts. Most evidence supporting rehabilitation-integrated DBS originates from specialized centers with multidisciplinary teams, which may not reflect real-world access in low- and middle-income settings.

Wearable sensor data show that DBS reduces gait irregularity and is associated with improved responsiveness to physiotherapy, especially in balance and lower-limb programs [76]. Remote monitoring studies similarly indicate that periods of lower subthalamic beta activity may correspond to more favorable windows for gait rehabilitation [35]. Balance and postural control remain only partially responsive to DBS alone, reinforcing the need for targeted rehabilitation strategies [77].

A recent Delphi consensus supports early, task-specific physiotherapy after DBS, highlighting benefits in gait amplitude, dual-task performance, and postural stability when training is delivered once stimulation parameters are clinically optimized [34]. Case-based evidence also suggests that combining DBS with intensive, goal-directed rehabilitation can produce additional gains in functional mobility and daily activities beyond stimulation alone [78]. Additional clinical data suggest that structured rehabilitation improves mobility and functional performance even years after DBS implantation [79].

Newer DBS technologies strengthen these clinical applications. Sensing-enabled and adaptive systems offer physiological markers that may help clinicians time therapy sessions to periods of optimal neural stability [32,80,81]. Neuromodulation-enhanced rehabilitation frameworks further propose that DBS may support adaptive plasticity [76,78,82]. Advances in functional mobility assessment techniques, such as integrated motion-capture and pressure-based gait analysis, may further enhance the ability to quantify rehabilitation response after DBS (Table 4) [83].

In this view, DBS may serve as a physiological facilitator of therapy-driven improvement under specific clinical and technological conditions. This role—sometimes described as a rehabilitation “amplifier”—remains conceptual, as confirmation of additive or synergistic effects requires mechanistic and controlled clinical studies.

Complementary rehabilitative technologies such as virtual reality may also enhance gait and balance training in DBS patients in selected phenotypes [84].

## 4. Limitations

This review is narrative rather than systematic, and not all relevant literature may have been captured. Heterogeneity in study design, imaging methods, and follow-up restricts comparability. Mechanistic insights derive disproportionately from PD studies.

Rehabilitation protocols after DBS remain highly heterogeneous, with limited evidence to guide optimal timing, intensity, or phenotype-specific adaptations.

An important limitation of the current DBS literature—and, by extension, of the present narrative review—is the limited representativeness of studied populations. Most mechanistic, imaging, and rehabilitation-related DBS evidence has been generated in high-income Western academic centers with access to advanced neurosurgical technologies, expert postoperative programming, and structured multidisciplinary rehabilitation. As a result, the generalizability of network-guided and precision-oriented DBS frameworks to broader global populations and diverse healthcare systems remains uncertain.

Differences in socioeconomic context, healthcare infrastructure, comorbidity burden, educational background, and access to rehabilitation services may influence baseline network organization, stimulation responsiveness, and functional recovery trajectories. Consequently, current precision-oriented DBS models should be interpreted as population-anchored rather than population-agnostic. Addressing demographic, geographic, and socioeconomic diversity represents a critical priority for future research aimed at validating and extending rehabilitation-integrated DBS frameworks.

## 5. Future Directions

Future progress in deep brain stimulation (DBS) will require integration of physiological biomarkers, individualized connectomic targeting, and adaptive neuromodulation strategies. Sensing-enabled systems may align stimulation and rehabilitation with neural states favorable for motor learning, while patient-specific pathway modeling could refine circuit selection across phenotypes. Real-world motor monitoring through wearable sensors may also help quantify long-term plasticity and guide rehabilitation dosing. Ultimately, mechanistically informed and controlled clinical trials are needed to determine how DBS-induced network stabilization interacts with structured rehabilitation to support durable functional recovery.

## 6. Conclusions

DBS has progressed from a focal intervention to a systems-level neuromodulation therapy informed by circuit physiology, imaging biomarkers, and technological innovation. Available evidence suggests that DBS modulates distributed networks, stabilizes motor output, and may create physiological conditions that support rehabilitation-driven improvement.

Technological developments, including directional leads, tractography-guided targeting, and adaptive systems, strengthen the interface between stimulation and therapy. Yet integrated frameworks linking neuromodulation with functional recovery remain nascent.

Overall, DBS may act as a physiological facilitator of rehabilitation under specific clinical, technological, and healthcare conditions; a conceptual model that requires further mechanistic refinement and controlled clinical validation.

## 7. Key Highlights

- Deep brain stimulation (DBS) enhances the stability of motor performance, creating favorable conditions for structured rehabilitation.
- Connectivity-informed targeting has been associated with improved clinical outcomes by aligning stimulation with patient-specific circuit architecture.
- Technological advances—including tractography-based planning, directional leads, and sensing-enabled systems—support more precise and individualized neuromodulation.
- Rehabilitation integrated with DBS may amplify functional gains by leveraging stabilized neural dynamics.
- Future frameworks will incorporate adaptive stimulation, biomarker-guided therapy, and real-world motor monitoring to optimize long-term functional restoration.

## Figures and Tables

**Table 1:** Clinical phenotypes, predominant network involvement, and rehabilitation considerations in DBS.

Clinical Phenotype (Examples)	Predominant Network Involvement	Rehabilitation Considerations
Tremor-dominant PD	Predominantly cerebellothalamic networks, with variable involvement of hyper-direct pathways	Tremor stabilization may facilitate fine motor retraining; task-specific upper-limb training is prioritized once oscillatory variability is reduced
Akinetic-rigid PD	Hyperdirect cortico–subthalamic and basal ganglia–thalamo–cortical networks	Gait initiation, rigidity reduction, and bradykinesia-oriented motor retraining may benefit from reduced beta variability during stimulation
Postural instability/gait disorder PD	Mixed motor and associative networks; frequent involvement of axial and postural control circuits	Balance and gait training often require individualized, multimodal strategies; response to rehabilitation remains heterogeneous
Classic ET	Predominantly cerebellothalamic networks (DRTT)	Tremor suppression enables fine motor and functional task retraining; rehabilitation impact varies with tremor regularization
ET-plus/cerebellar tremor variants	Cerebellothalamic networks with variable brainstem and cortical involvement	Rehabilitation response is highly variable; individualized task selection and adaptive training strategies are often required
Dystonic tremor	Mixed cerebellothalamic–pallidal-network involvement	Motor retraining often requires flexible, patient-specific approaches due to hybrid network dysfunction
Generalized or genetic dystonia	Pallidothalamic and sensorimotor networks with variable associative–limbic contribution	Functional gains may emerge gradually; rehabilitation strategies require long-term adaptation and close alignment with stimulation effects

Listed networks reflect predominant patterns reported in the literature rather than fixed or mutually exclusive circuit assignments. Overlap, mixed phenotypes, and atypical network configurations are common, highlighting the need for individualized interpretation of symptom severity and for tailoring stimulation and rehabilitation strategies. Abbreviations: PD—Parkinson’s disease; ET—essential tremor; DRTT—dentato-rubro-thalamic tract.

**Table 2:** Multiscale biological mechanisms of DBS and their relevance for rehabilitation.

Mechanistic Level	Key Mechanisms	Rehabilitation Relevance
Microscale (neuronal)	Axonal activation; somatic suppression; altered firing patterns	Stabilizes motor output and supports consistent performance during training
Mesoscale (oscillatory)	Beta suppression; ERNA; short-latency entrainment	Enhances motor learning and improves within-session stability
Macroscale (network)	Modulation of hyperdirect, cerebellothalamic, and pallidothalamic circuits	Aligns stimulation with gait, fine-motor, and functional rehabilitation goals
Non-neuronal/ molecular	Astrocytic modulation, adenosine release, trophic signaling	Supports adaptive plasticity and learning-dependent improvement

Multiscale neuronal, oscillatory, network-level, and non-neuronal mechanisms of DBS relevant to functional rehabilitation, illustrating how these mechanisms relate to rehabilitative potential.

**Table 3:** Technological Innovations in DBS and Their Functional/Rehabilitative Implications.

Technology	Core Feature	Clinical/Rehabilitation Relevance
High-field MRI + tractography	Patient-specific visualization of relevant pathways	Improves targeting precision and reduces side effects, supporting alignment of stimulation with functional goals
Directional leads	Current steering toward therapeutic pathways	Widens therapeutic window; improves stability for high-intensity rehabilitation
Sensing-enabled DBS	Continuous monitoring of physiological biomarkers	Enables objective programming and reduces variability affecting therapy performance
Adaptive DBS (closed-loop)	Stimulation delivered when biomarkers exceed thresholds	Improves gait/tremor stability and supports timing of rehabilitation tasks
Wearable motor sensors	Continuous monitoring of gait, tremor, bradykinesia	Enables therapy personalization and home-based training
Connectomic programming platforms	Lead reconstructions + pathway-activation modeling	Supports individualized programming based on patient-specific networks

Key DBS technologies and their clinical and rehabilitation implications.

**Table 4:** Core rehabilitation domains and functional goals following DBS in movement disorders.

Domain	Main Goals
Physiotherapy	Improve gait, balance, amplitude, and dual-task performance
Occupational therapy	Enhance dexterity, handwriting, and ADLs
Speech–language therapy	Improve articulation, phonation, and intelligibility
Cognitive–behavioral support	Maintain executive functioning, mood, and therapy engagement
Home/community training	Promote task-specific practice and generalization to daily life

Primary rehabilitation domains and goals following DBS. These domains outline the therapeutic framework typically integrated with neuromodulation-based care. Examples are drawn from rehabilitation-related studies in Parkinson’s disease, essential tremor, and dystonia, as discussed in Section 3.7 [69,70,71,72]. They are intended to illustrate typical functional targets rather than prescribe device-specific protocols. Access to these rehabilitation domains varies substantially across healthcare systems and socioeconomic contexts, and most supporting evidence derives from specialized centers with multidisciplinary resources.

## Data Availability

No new data were generated or analyzed in this study. All data discussed in this review are derived from previously published sources.

## Abbreviations

DBS	Deep brain stimulation
DRTT	Dentato–rubro–thalamic tract
ET	Essential tremor
ERNA	Evoked resonant neural activity
fMRI	Functional magnetic resonance imaging
GA1	Glutaric aciduria type I
GPi	Globus pallidus internus
KMT2B	Lysine methyltransferase 2B
MRI	Magnetic resonance imaging
PD	Parkinson’s disease
PET	Positron emission tomography
PIGD	Postural instability/gait disorder
PSA	Posterior subthalamic area
SPECT	Single-photon emission computed tomography
STN	Subthalamic nucleus
VIM	Ventral intermediate nucleus
ZI	Zona incerta
